# High Voltages in Sliding Water Drops

**DOI:** 10.1021/acs.jpclett.3c02864

**Published:** 2023-12-05

**Authors:** Pravash Bista, Aaron D. Ratschow, Hans-Jürgen Butt, Stefan A. L. Weber

**Affiliations:** † Max Planck Institute for Polymer Research, Ackermannweg 10, Mainz 55128, Germany; ‡ Institute for Nano- and Microfluidics, TU Darmstadt, Peter-Grünberg-Strasse 10, Darmstadt 64289, Germany; ¶ Department of Physics, Johannes Gutenberg University, Staudingerweg 10, Mainz 55128, Germany

## Abstract

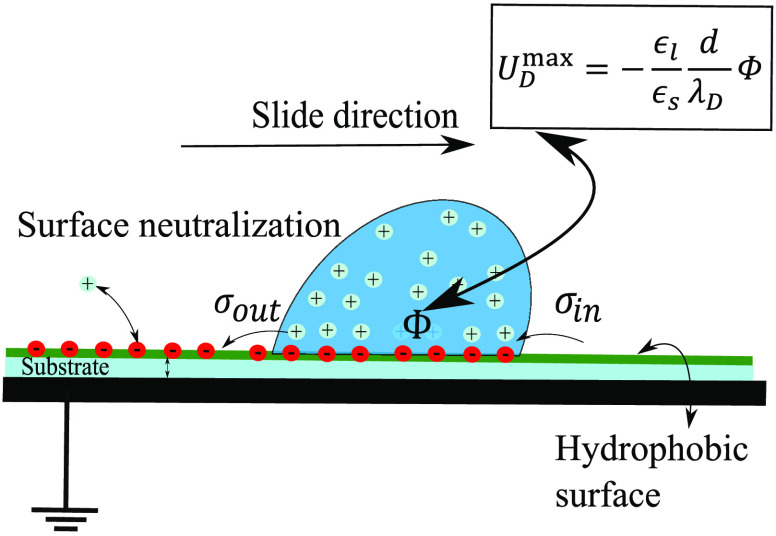

Water drops on insulating hydrophobic substrates can generate electric potentials of kilovolts upon sliding for a few centimeters. We show that the drop saturation voltage corresponds to an amplified value of the solid–liquid surface potential at the substrate. The amplification is given by the substrate geometry, the drop and substrate dielectric properties, and the Debye length within the liquid. Next to enabling an easy and low-cost way to measure surface- and zeta- potentials, the high drop voltages have implications for energy harvesting, droplet microfluidics, and electrostatic discharge protection.

Spontaneous charging in moving drops is commonly observed in micropipetting,^1,2^ aerosolizing,^3^ bouncing,^4−9^ squeezing,^10^ and sliding of drops (slide electrification).^11−15^ Many studies have highlighted the potential of this charge separation process in energy harvesting^16−27^ or sensing.^28^ Although this phenomenon has been observed qualitatively for decades,^6^ a quantitative understanding of the physical process would vastly expand applicability.

One simple way to study slide electrification is to measure the discharge current of a drop after sliding down a tilted hydrophobic plate.^13,14,29,30^ Using this method, we observed that the drop charge saturates after a few cm. We furthermore found a dependence of the drop charge on the surface chemistry^31^ and a drop number dependence during a sequence of drops.^13^

The exact mechanism of the charge transfer is still under investigation. Next to electron transfer,^10,14,29^ the phenomenon is commonly attributed to ionic charges.^2,32−36^ In water or high-dielectric liquids, most solid surfaces are charged. These surface charges form spontaneously, e.g., by the adsorption of ions from solution, by protonation or deprotonation of surface groups, or by the preferential dissolution of ions, leading to the formation of an electrostatic double layer (EDL).^37,38^ Sosa et al. have shown that contact electrification is correlated to the zeta potential, pH, and salt concatenation of the liquid.^39^ Thus, previous models were based on the assumption that some of the charge from an EDL is left behind on the solid surface as the contact line moves.^13^ Recently, this charge transfer mechanism at receding contact lines and its parametric dependencies were described theoretically.^40^

The solid–liquid surface charging is commonly described by means of the surface potential, Φ, which is the electrostatic potential at the transition between immobilized countercharges in the so-called Stern layer and the diffuse Debye layer. Because of the high capacitance of the Stern layer,^41^ the often cited zeta potential at the shear plane and the surface potential are almost indistinguishable for Debye lengths above ∼1 nm. This surface potential is one of the fundamental properties in colloid and interface science. It determines the stability of dispersions and emulsions, causes electrokinetic phenomena and corrosion, and influences catalytic activity, contact angles, and the thickness of thin liquid films.^42^ Moreover, the surface potential is a fundamental property of biological and technical membranes. Still, it is difficult to measure the electric properties of solid–liquid interfaces. For dispersed particles, zeta potentials can be measured by electrophoresis or electroacoustics, and for certain charging mechanisms, surface charge densities can be determined by titration. For planar surfaces, streaming potentials can be measured or the surface potential can indirectly be determined from AFM force measurements. However, these methods are often unprecise and notoriously unreliable and have to rely on many assumptions.

Here, we investigate the connection between the saturation voltage or charge acquired by sliding drops and the physicochemical surface properties, such as the surface potential. To this end, we developed a method to measure the capacitance and potential of drops on hydrophobized glass substrates. These measurements revealed drop voltages exceeding 1–3 kV, with a drop capacitance of *C*_D_ = 1.2 ± 0.1 pF, which is the equivalent capacitance of the drop–substrate system. We rationalize the high saturation voltages by considering the electrostatic fields at the solid–liquid interface, revealing the connection between the drop charge and the surface potential. Thus, by measuring the saturated drop potential, we can determine the surface potential at the solid–water interface.

Charge and voltage measurements were performed for drops sliding down an inclined glass substrate (soda lime glass with 35 nm Au sputtered on the backside) with a tilt angle of 50° and thickness of *d* = 1 mm on a grounded metal plate (Figure.1). The substrates were hydrophobized with (trichloro(1H,1H,2H,2H-perfluorooctyl)silane, PFOTS, via chemical vapor deposition. Prior to the experiments, the substrate was neutralized using an ionizing air blower for 2 min. The experiments were done under ambient conditions (temperature: 21 ± 1 °C; humidity: 35–55%). The charge accumulated by a neutral, deionized water drop (*V* = 45 μL, Sartorius Arium Pro VF, 18.2 MΩ resistivity, in equilibrium with atmospheric CO_2_, pH ≈ 6) sliding on an initially neutral hydrophobic substrate was measured using a gold-plated metal electrode connected to a subfemtoampere current amplifier (rise time: 0.7–1.8 μs, FEMTO DLPCA-200, Berlin, Germany; details in the Supporting Information S1).

**Figure 1:**
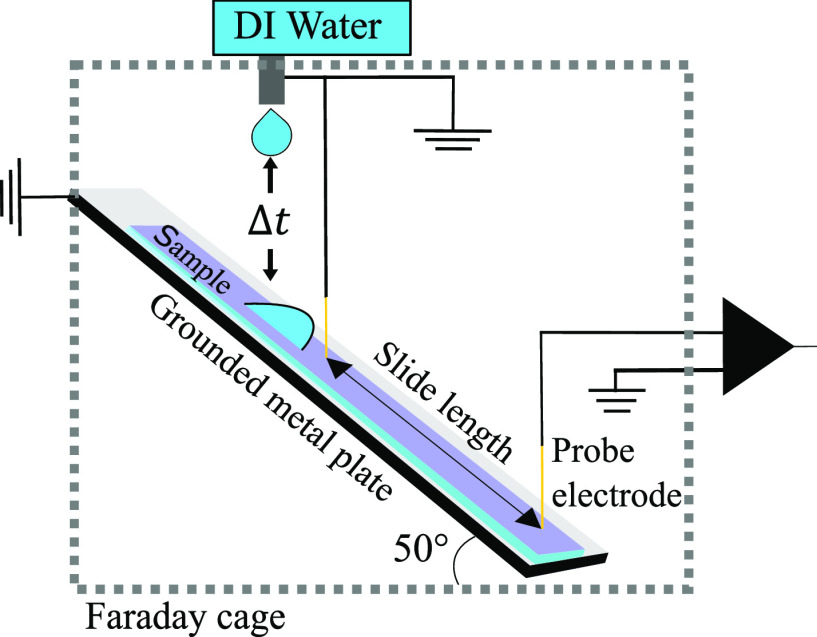
Illustration of the experimental setup (detail in section S1).

We measured the drop charge and voltage at different slide lengths. The drop charge and voltage values as a function of slide length for different drops are shown in Figure 2a, which we call drop charge traces. The first drop sliding on a neutralized surface accumulated a maximum charge of 1.35 ± 0.03 nC over a distance of *L*_sat_ = 7.0 ± 0.3 mm. In contrast to the drop charge, a reliable direct measurement of the drop voltage at such low charge values is more difficult. It requires a voltmeter with a high input impedance and low stray capacitance. We addressed this challenge from two sides: In a first approach, we measured the electrostatic drop–substrate capacitance using a static drop with a bottom electrode underneath the substrate (see Figure S3a). Here, we applied an external voltage (*V*) to the drop and measured the image charge on the bottom electrode (*Q*) (section S1.4,^43,44^). This way, we measured a drop capacitance of *C*_D_ = *Q*/*V* = 1.22 ± 0.02 pF, which is in good agreement with the theoretically estimated value (section S 1.4). From here, we calculated the drop voltage via *U*_D_^1^ = *Q*_D_^1^/*C*_D_ = 1.1 kV.

**Figure 2:**
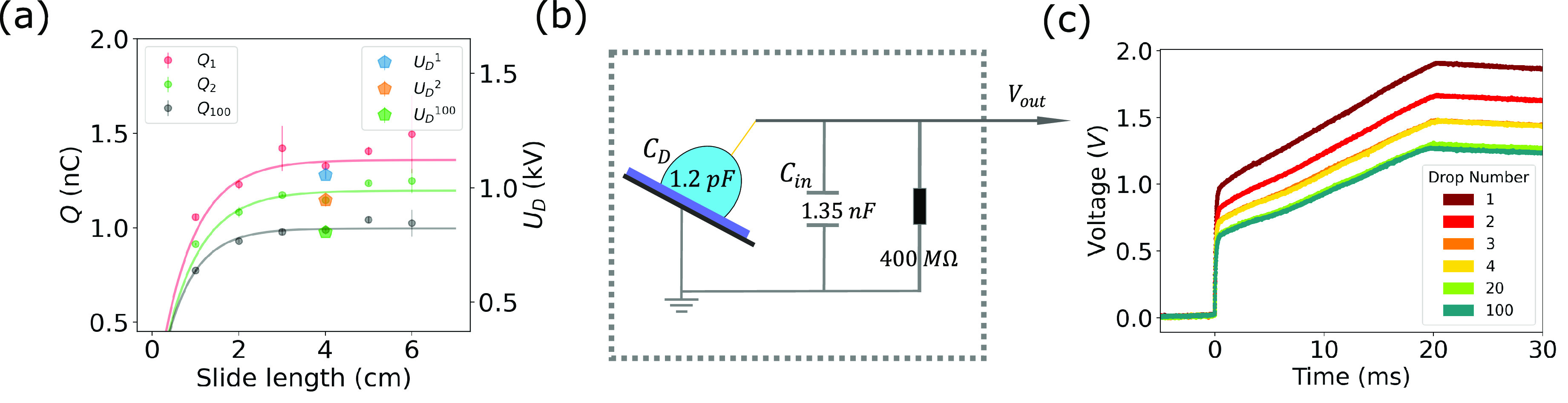
(a) Drop charge versus slide distance of the 1st, 2nd, and 100th drop plotted with an exponential fit to obtain characteristic saturation length *L*_sat_ = 7.0 ± 0.3 mm. (b) Setup to measure the drop voltage with an input capacitance of *C*_in_ = 1.35 nF (cables, capacitor, and DAQ input capacitance) and 400 MΩ resistor. (c) Voltage measured at *C*_in_. The first jump to 0.98 V is due to the drop discharge; the subsequent linear increase comes from the ongoing charge separation during drop sliding. We can calculate the initial drop voltage using the scaling factor *C*_in_*/C*_D_ = 1125 to be 1.1 kV.

In a second approach, we measured the drop voltage using a capacitive voltage divider. Here, we used a gold-plated metal electrode connected to an input capacitance of *C*_in_ = 1.35 nF to measure the drop potential, Figure 2b (details are provided in section S1.3). Once in contact with the metal electrode, the drop discharged into the input capacitance *C*_in_ until the voltages were equalized. The measured voltage depends on the capacitance ratio between *C*_in_ and *C*_D_. The voltage vs time for the first drop (brown curve in Figure 2c) shows a voltage jump within the first millisecond due to the redistribution of the drop charge across the total capacitance. It is followed by a linear voltage increase due to the ongoing charge separation at the moving contact line. The current resulting from ongoing charge separation at the receding contact line can be estimated as , which yields a value of 70–80 nA. This estimation closely matches the values measured in a previous study^40^ and can also be seen in the drop discharge current in Figure S1c. After the drop has passed the electrode, the voltage signal showed a slow decrease. To enable measurements on a sequence of drops, we allowed the system to discharge over an additional 400 MΩ resistor. By using the capacitance ratio of (*C*_in_ + *C*_D_)/*C*_D_ ≈ *C*_in_/*C*_D_ for *C*_in_ ≫ *C*_D_, we estimated the initial voltage within *C*_D_ prior to its contact with *C*_in_. These measurements yielded a drop voltage of 1.10 ± 0.02 kV, consistent with the previous capacitance measurement.

By comparing the voltage-to-drop charge data collected with the current amplifier on the same substrate at different slide distances, we can estimate the average drop capacitance by using the drop charge and voltage. This estimation yields *C*_D_ = 1.2 ± 0.1 pF, which is consistent with the value measured for a static drop (section S1.4). Both methods show that the sliding drops spontaneously charge up to a significant voltage on the order of kV. At saturation, the drops carry an electrostatic energy of *W*_D_ = 0.8 μJ per drop.

The charge separation process seems to happen spontaneously with a strong electrostatic potential. To understand the process, we consider a single drop sliding on a neutral substrate. Because of charge conservation and the insulating nature of both the substrate and the surrounding air, any change in total drop charge, d*Q*, is the result of surface charges, σ_out_, leaving the drop at the receding contact line. Thus, the change in drop charge of a drop with width *w*, sliding a distance of *dx*, at location *x* can be expressed as
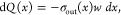
1

It is well-known that solid surfaces in contact with liquid water acquire a net charge σ_SL_ and form an EDL by attracting a diffuse layer of counterions. The characteristic thickness of this diffuse layer is called the Debye length λ_D_. The surface charge can be caused by surface chemistry processes and/or specific ion adsorption.^38^ The fundamental mechanism of charge separation at receding contact lines is the dewetting of bound surface charges from the EDL.^40^ It is thus reasonable to assume that a fraction 0 ≤ α ≤ 1 of the surface charge is deposited by the drop.^13^ Assuming α to be constant, as valid for the drop velocity range during the experiments,^40^ we can write
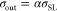
2To quantify σ_SL_, we consider Gauss’s law at the solid–liquid interface
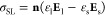
3with the normal vector of the interface, **n**, permittivity ε, and electric field **E** in the liquid (l) and solid (s). The electric field in the liquid, **E**_l_, is governed by the EDL. For moderate surface potentials, Φ < *kT*/*e*, the electric field in the liquid scales like **nE**_l_ = Φ/λ_D_. Here, *k* is the Boltzmann constant, *T* is the temperature, *e* is the unit charge, and λ_D_ is the Debye length. For water with a monovalent salt at concentration *c*_0_, the latter is given by . The electric field in the substrate, **E**_s_, is determined by the potential difference between the drop with charge *Q* and the grounded plate under the substrate with thickness *d* (Figure 3a) via **nE**_s_ = −*U*_D_/*d*.

**Figure 3:**
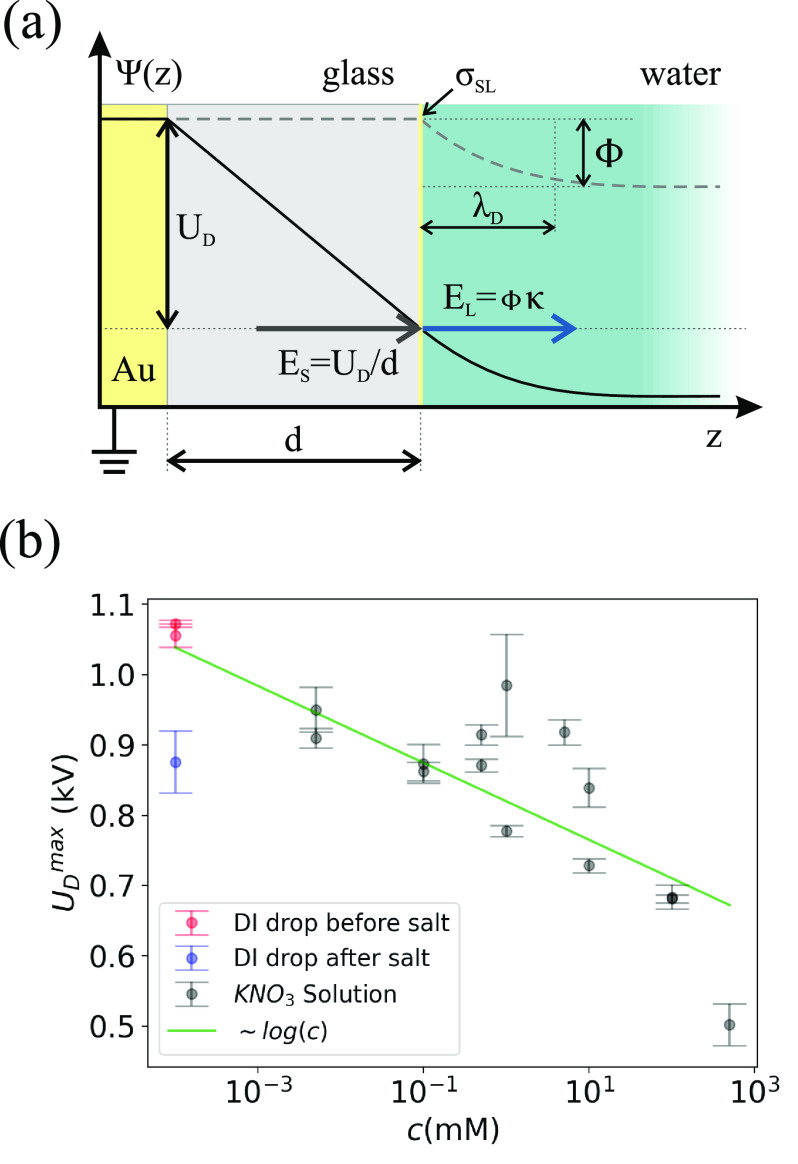
(a) Electrostatic potential Ψ(*z*) between the drop and metal electrode. For an uncharged drop, the potential is flat throughout the substrate (dashed line). The surface charge density at the solid–liquid interface, σ_SL_, results from a discontinuity in the dielectric displacement. For a charged drop (solid line), there is an electric field present in the substrate, reducing the jump in the dielectric displacement and thereby the effective surface charge density. (b) Voltage, *U*_D_^max^, with increasing KNO_3_ concentration. The blue data point shows a measurement using a deionized (DI) water drop after a series of measurements with salty drops, indicating potential irreversible changes at the solid-liquid interface.

Overall, the interfacial Gauss’s law yields
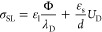
4This relationship is general as it only contains the assumption of a moderate surface potential. However, eq 4 has two free variables: solid–liquid surface charge σ_SL_ and surface potential Φ.

Generally, a change in the drop potential could also shift the chemical equilibrium at the solid–liquid interface, thus generating a nonlinear response of both σ_SL_ and Φ to the drop voltage *U*_D_.^45,46^ This potentially complex process can be linearized for an approximately constant voltage across the diffuse layer and, thus, an approximately constant surface potential. In this linearized case, the drop charge would saturate as a function of distance, in agreement with our observations (Figure 2a). Therefore, the linearization adequately captures the observed effects.

We assume that the voltage across the EDL is always identical to the surface potential Φ of a neutral drop. Using the surface charge within the drop (eq 4), we get
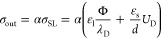
5Inserting into eq 1, we arrive at the model equation for the drop charge
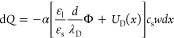
6Here, *c*_s_ = *ε*_s_/*d* is the specific substrate capacitance. We can immediately see that the drop charge will be stationary (d*Q* = 0) at a maximum drop voltage of
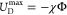
7
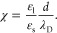
8Thus, the saturation voltage, *U*_D_^max^, is an electrostatically amplified value of the surface potential Φ. Here, we defined the amplification factor χ that is proportional to the ratio of the dielectric permittivities of the liquid and substrate and the ratio of substrate thickness *d* and Debye length λ_D_. Using values from the experiments (ε_s_ = 7ε_0_, ε_l_ = 80ε_0_, *d* = 1 mm, and λ_D_ ≈ 400 nm), we get a value of χ = 28571. With the measured saturation value of *U*_D_^max^ = 1.1 kV, we can calculate Φ ≈ −38.0 mV, which is close to the zeta potential reported in literature.^47^ No significant difference in measured voltage was observed between 5% and 88% humidity. By rearranging eq 7 and using the drop–substrate capacitance *C*_D_ = ε_s_*A*/*d*, we can find a similar relationship for the drop charge
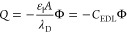
9where *C*_EDL_ is the EDL capacitance within the drop.

Thus, by measuring the saturated drop charge or voltage after a sufficiently long slide distance, the surface potential of the solid–liquid surface can be determined. The proposed method is independent of the specific transfer coefficient α. Once the drop has reached its steady-state potential, no charge is transferred to the substrate. The transfer coefficient only determines the required characteristic slide distance required to reach the stated state.

To understand the role of the charge transfer coefficient α, we can rearrange eq 6 by using the drop capacitance *C*_D_ = ε_s_π*w*^2^/(4*d*) = ε_s_*A*/*d* and the maximum drop charge *Q*_D_^max^ = −χΦ*C*_D_
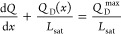
10Here,  is the characteristic saturation length of the drop charge. With the measured saturation length of *L*_sat_ = 7.0 ± 0.3 mm and a drop width of *w* = 5 mm, we can estimate the charge transfer coefficient to be α ≈ 0.5.

Equations 7 and 8 predict that the saturation drop voltage is proportional to the substrate thickness, *d*. We repeated our measurements on a 3 mm thick glass substrate and observed a roughly 3-fold increase in the drop voltage to 3.8 ± 0.1 kV (Φ ≈ −44 mV). Interestingly, the measured drop charge remained almost constant. The voltage increase is the result of the lower capacitance on the thicker substrate. Note that for substrate thicknesses exceeding the drop size, *d* ≫ *w*, the drop capacitance takes the form of a charged sphere between two dielectrics and the model assumptions no longer apply.

Similarly, the theory predicts a dependence of the saturation voltage on the Debye length, at least for moderate salt concentrations within the Debye–Hückel approximation. To test this dependence, we performed experiments on the initial 1 mm thick substrate using drops with different concentrations of KNO_3_. The voltage measurements revealed a decreasing voltage with increasing ion concentration (Figure 3b), which is consistent with literature.^48^ In Figure 3b, the red dots represent the maximum value of *U*_D_ on the pristine surface without salt. We measured the voltage generated by the droplets while increasing the salt concentration and washed the surface between each measurement. We observed that the voltage decreased with increasing concentration, *c*. Upon repeating the measurement with DI water (blue dot), we found that there has been a permanent change to the surface.

So far, we have assumed that the Φ potential is independent of the salt concentration, *c*. It is well-known that at low potentials or high ion concentration Φ and λ_D_ are approximately proportional to *c*^–1/2^,^49^ making the product Φ/λ_D_ independent of *c*. At higher surface potentials (Φ ≫ *k*_B_*T*/*e*), a more general approximation is given by Φ ∼ log(λ_D_).^49^ The saturation voltage would thus be proportional to log(*c*) (*U*_D_^max^ ∼ Φ ∼ log(*c*)), which could explain the observed concentration dependence.

Our findings have immediate implications for energy harvesting from sliding drops. Previous studies reported rather low efficiencies of around 1%.^23,50^ Our theoretical analysis reveals that the saturation voltage *U*_D_^max^ increases proportionally to the substrate thickness (eq 7), while the saturation charge *Q* only depends on the wetted area and the EDL properties (eq 9). Thus, the total drop energy *W*_D_ = (1/2)*QU*_D_ increases linearly with increasing substrate thickness. In the present case, the drop charge saturates after sliding a height difference of Δ*z* = 3 cm, losing a potential energy of *W*_G_ = *mg*Δ*z* = 13 μJ. The electrical energy on a 1 mm thick substrate is *W*_D_ = 0.8 μJ, yielding an energy harvesting efficiency of 6%. By increasing the substrate thickness to 3 mm, the efficiency increases to 18%. Here, the upper limit is that the drops get stuck in their own electrostatic field. Thus, to optimize this process, a balance between droplet motion and energy harvesting has to be found.

To harvest the energy stored in the drops, it might be useful to have a continuous sequence of drops. When we let multiple drops run down the surface at a drop interval Δ*t* = 1.8 ± 0.2 s, the drop voltage vs drop number measured at a slide length of 5 cm showed a rapid decrease from 1100 to 850 V (Figure 4 dots) within the first ten drops. We repeated these measurements at different slide lengths and plotted the drop charge traces as a function of slide length for the 1st, 2nd, and 100th drop (Figure 2a). Subsequent drops saturated at lower voltages until reaching a stable value around 850 V. For subsequent drops, the saturation length increased because the surface was already partially charged from previous drops, reducing the charge transfer.

**Figure 4:**
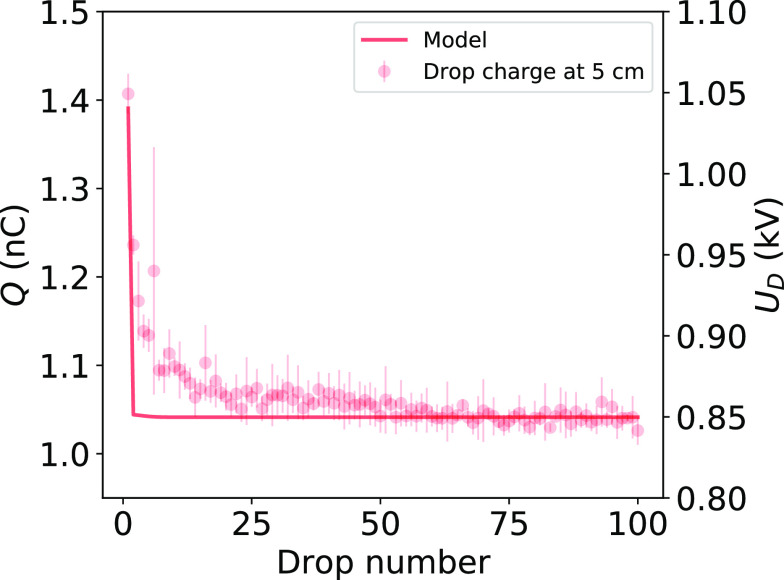
Measured drop charge on PFOTS (dots) and numerical simulation (line) using the model. Parameters used for the simulation are Δ*t* = 1.8 s, *C*_D_ = 1.2 pF, Φ = −40 mV, and τ = 2.3 s.

Our measurements and previous works suggest that the surface charge on the dry substrate decays with a characteristic time scale τ (section S2.2) on the order of 1–100 s^13^ through nonzero substrate or surface conductivity^51−53^ or by neutralization through ionic species in the ambient air.^54^ To account for these surface charges in the charge balance, we can modify eq 6 for the *n*th drop at position *x*
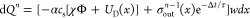
11where Δ*t* is the time in between two drops and σ_out_^0^ = 0 for initially uncharged substrates. Using this model in a numerical simulation that also takes into account the additional surface charge deposited during the electrode discharge (section S2.1), we find good agreement between simulation and experiment (Figure 4). Deviations from the model might be explained by a change in velocity, caused by electrostatic forces between the drop and surface charges.^15^ Furthermore, the observed behavior might be caused by the substrate polarization. To increase the energy harvesting efficiency in sequences of drops, substrates with a fast decay of surface charge are advantageous.

To conclude, drops sliding over hydrophobic insulating surfaces acquire voltages higher than 1 kV over a distance of a couple of centimeters. The measured saturation drop voltage represents an amplified value of the surface potential at the solid–liquid interface. The amplification originates from the electrostatic potential landscape at this interface. The simple electrostatic model derived in this Letter quantitatively captures the experimentally observed physics.

The evidence of kV potentials in sliding drops together with our model description has major implications for energy applications as it enables optimization of the energy harvesting efficiency. The high voltages in moving drops could destroy delicate structures, for example, in microelectronics. Furthermore, the discovery and theoretical description of the relationship between the measured saturation voltage *U*_D_ and the surface potential could spark a new research field by enabling low-cost surface and zeta potential measurements on flat surfaces.

## Supporting Information Available

Experimental procedure with sample preparation, drop charge measurement, voltage measurement setup and results, capacitance measurement of a static drop, slide electrification theory with numerical simulation, simulation results, estimation of surface discharge time, and surface potential at different humidity (PDF)
Transparent Peer Review report available (PDF)
