# Supplementary material for: High Voltages in Sliding Water Drops
Source: J Phys Chem Lett. 2023 Dec 5;14(49):11110–6. doi: 10.1021/acs.jpclett.3c02864 (PMC10726385; doi:10.1021/acs.jpclett.3c02864)
Supplement: Supplementary file 1 — jz3c02864_si_001.pdf [file jz3c02864_si_001.pdf]

High Voltages in Sliding Water Drops

Pravash Bista ,[a][†] Aaron D. Ratschow [a] ,[‡] Hans-Jürgen Butt,[†] and Stefan A. L. Weber[*,†,¶]

† *Max Planck Institute for Polymer Research, Ackermannweg 10, 55128 Mainz, Germany*

‡ *Institute for Nano- and Microfluidics, TU Darmstadt, Peter-Grünberg-Str. 10, 64289 Darmstadt, Germany*

¶ *Department of Physics, Johannes Gutenberg University, Staudingerweg 10, 55128 Mainz, Germany*

E-mail: webers@mpip-mainz.mpg.de

[a]P.B. and A.D.R. contributed equally to this work.

S1 Experimental Procedure

S1.1 Sample preparation

The substrates are microscope glass slides (Menzel glass, Thermo Fischer) coated with a 35 nm thick layer of gold on the back side. Before hydrophobizing the surface, the glass slides were cleaned with acetone and ethanol and treated in an oxygen plasma cleaner for 10 minutes at 300 W power (Diener Electronics Plasma surface: Femto BLS, Ebhausen, Germany), to activate the surface and to remove all organic compounds. We used chemical vapor deposition to coat the front side of glass substrates with (trichloro(1H,1H,2H,2H-perfluorooctyl)silane (PFOTS, Sigma-Aldrich Chemie GmbH, Eschenstrasse 5, 82024 Taufkirchen Deutschland Germany). We placed the substrates and a 1 mL vial of PFOTS with a magnetic stir bar in a vacuum desiccator. A pump evacuated the desiccator to a pressure of 100 mbar,

which vaporized the PFOTS. Then the chamber was left on a stir plate for 30 minutes to complete the silanization process. The motion of the magnetic stir bar provided airflow within the desiccator for more homogeneous deposition. The PFOTS surface had advancing and receding contact angles of (107 ± 2)° and (89 ± 3)°, respectively.

S1.2 Drop charge measurement

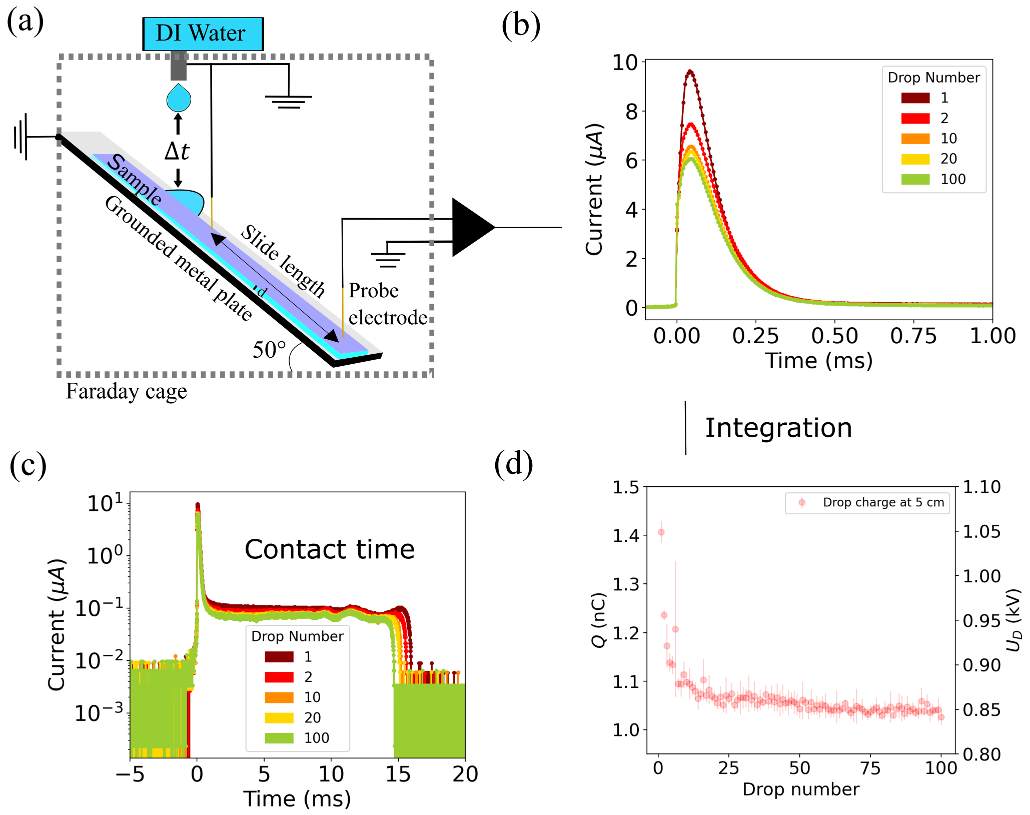

Figure S1: (a) Illustration of experimental setup, (b) current signal measured as the probe electrode discharges the sliding drop , (c) drop-surface contact time t_c, and (d) drop charge estimated by integrating the initial peak of around 0.5 ms.

Our experimental setup is shown in figure S1(a). The experiments were done under ambient conditions (temperature: 21 ± 1°C, humidity: 30-50%), where minimal influence of humidity and temperature has been reported.[1] We placed the PFOTS-coated substrates on an inclined grounded plate with a tilt angle of 50° (unless otherwise noted). We neutralized any surface charge using an ionizing air blower (IAB, Simco-Ion Aerostat PC Ionizing air

blower) for 2 minutes (air ion concentration to 300000 $ions/cm^3$, measured with an Ionometer, IM806v3, 66687 Wadern, Germany), and waited some minutes until ions in the air dissipated and an equilibrium air ion concentration (200-400 $ions/cm^3$) was reached. Next, a peristaltic pump (Gilson Minipuls 3, Wisconson, USA) was used to pump deionized water (Sartorius Arium Pro VF, 18.2 MΩ resistivity, Germany, pH= 6.4 ± 0.3) into the grounded metallic syringe (diameter 2 mm), that produced water drops of volume 45 μL. As we showed in our previous work (Supplemental information in[2]), the falling drops were neutral. It is well-known that DI water quickly dissolves CO_2 from the atmosphere,[3,4] leading to a pH of around 6. Dissolved CO_2 can introduce HCO_3^- ions in the drop in addition to OH^- and H_3O^+.[3]

We chose a drop interval time of $\Delta t = 1.8 \pm 0.2\ s$ between drops for all experiments (unless otherwise noted). The drops fell from the height of (0.5 ± 0.2) cm and slid for (1.0 ± 0.2) cm to a grounding metal electrode, where they were neutralized to begin the slide electrification experiment. We choose a thin metal grounding wire so that the drop motion was not influenced. We have to follow this procedure because drops can already accumulate charges through the spreading and retraction of the drop on the surface during impact.[2] The grounding electrode marked the starting point of the slide experiment ($L = 0$). Drop widths (w) and lengths (l) were measured using a calibrated telecentric high-speed camera. From previous studies of our group, it was found: for 45 μL drops: $w = 5$ mm, and $l = 6.7$ mm.[5] After getting neutralized at the grounding electrode, the drop then slid a known distance (accuracy of $L \pm 0.2$ cm) to a gold plated metal probe electrode. A laser trigger system consisting of a 657 nm laser diode (CPS186, Thorlabs, United States) and a laser detector were placed 1 cm before the probe electrode and served as a trigger for data acquisition.

S1.3 Current and voltage measurement

When the charged droplet touched the probe electrode, the drops were either discharged via a a sub-femtoampere current amplifier (current measurement, FEMTO DLPCA-200 ,

Berlin, Germany; rise time: 0.7-1.8 μs) or directly on a capacitor (voltage measurement). We used a national instrument data acquisition board (NI USB-6366 x-Series) to record the current or voltage signal. The current measurement yielded signals as shown in figure S1b. To calculate the accumulated drop charge over a chosen slide length, we integrated the initial peak of the measured current signal, which was within the first 0.5 ms (S1 b) of drop probe contact.

To measure the voltage of sliding drops, we connected a 1 nF capacitor in parallel with the input of the NI card. The input has a high input resistance of 100 GΩ and an input capacitance of 100 pF. Together with the cable, we measured a total capacitance of the system of 1.35 nF using an LCR meter and also using the discharge time and resistor (Fig.S2 d). An additional resistor with 400 MΩ ensures that the capacitors can discharge in between drops, while keeping the voltage stable during the drop contact. The schematic of the voltage probe is shown in figure S2(a). The voltage in capacitor C_D before the contact with the probe capacitor, with capacitance C_{in}, is denoted as U_D. The charge in C_D is represented as Q. After the contact, the total charge equilibrates between the two parallel capacitors, and both attain the same voltage. The total charge in this situation is given by $Q = C_{\text{total}}U_{in} = (C_D + C_{in})U_{in}$. Solving this equation yields the voltage ratio: $U_D = \frac{C_{in}+C_D}{C_D}U_{in}$. From the measured voltage, we calculated the initial drop voltage using the ratio of two capacitance $U_D = \frac{C_{in}+C_D}{C_D}U_{in}$. Figure S2(b) shows the measured voltage curves. The high voltage measured with this technique is consistent with the voltage we estimated using the capacitance method. For the simple estimation of the capacitance (C_D) of subsequent drops, we used the the total charge (Q) measured using the transimpedance amplifier and drop voltage (U_D). Figure S2(c) shows that the capacitance for each drop is in accordance with the value we measured with capacitance method.

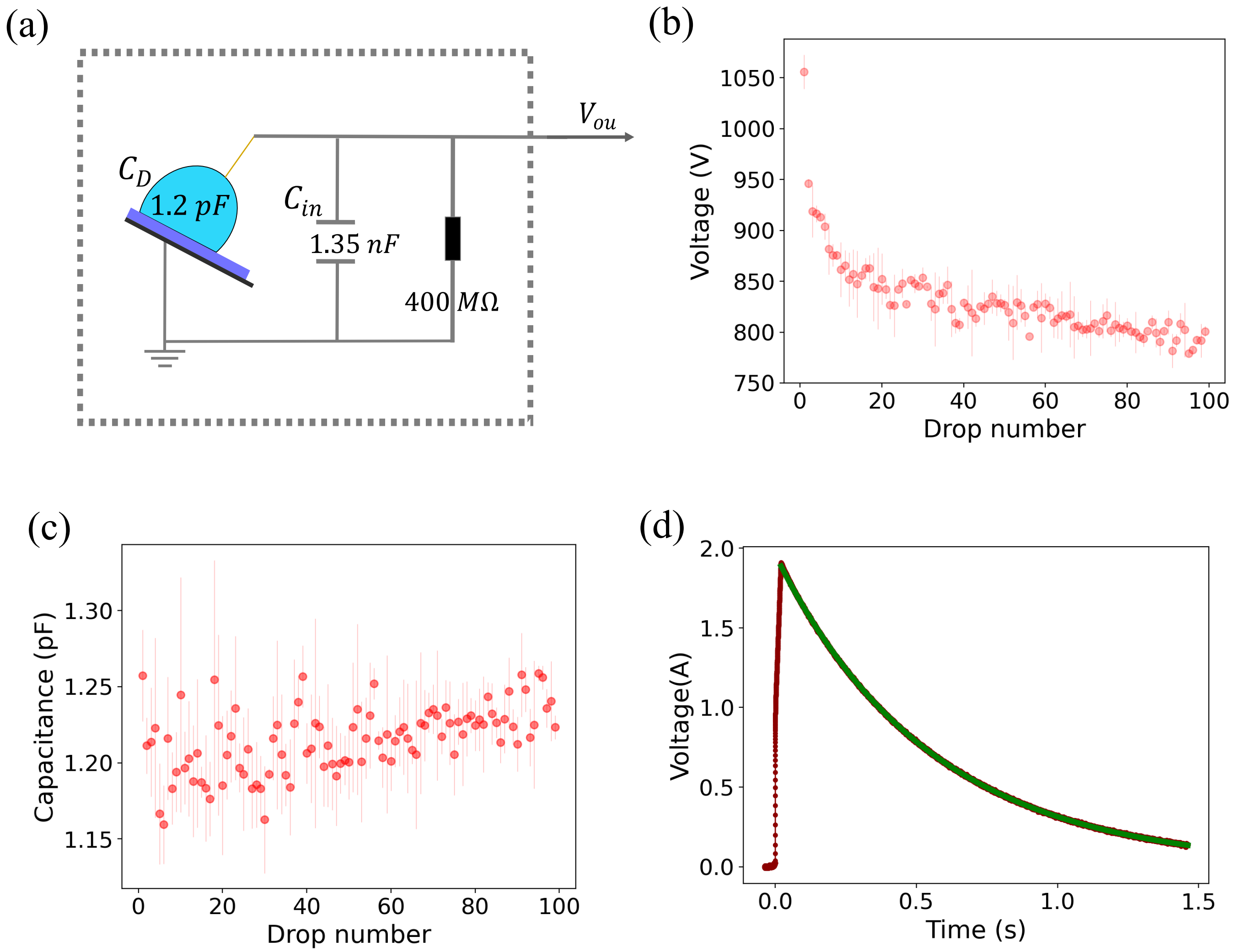

Figure S2: (a) Setup to measure the high drop voltage using capacitors in parallel. (b) Drop voltage measured for successive drops. (c) Capacitance estimated using the drop charge and drop voltage ($C_D = \frac{Q}{U_D}$). (d) Discharge time of the input capacitor via 400 MΩ resistor.

S1.4 Capacitance measurements

To measure the capacitance of the drop-surface system, we conducted an experiment where we charged up the capacitor externally using a potential, V, and measured the charge, Q, in the capacitor system. Using Q and V for 45 μL drop , we estimated an average C_D=1.22 ± 0.02 pF and C_D=0.47 ± 0.09 pF for 1 and 3 mm glass substrate respectively. Another way to estimate the capacitance is by using a plate capacitor approximation. We used the

substrate dielectric $\epsilon_r \approx 7.0$ for soda lime glass, drop base area ($A = \frac{\pi \cdot l \cdot w}{4}$), and the glass thickness d in the following equation

$$C = \epsilon_0 \epsilon_r \frac{A}{d}, \tag{S1}$$

which yields the approximated capacitance of our system $\approx$ 1.38 pF and 0.48 pF for 1 and 3 mm substrate, respectively. The slight deviation from the measurement should be caused by the difference between sessile and moving drops. We further want to state that the measurement of such a small capacitance is highly susceptible to error. Thus the good agreement of mesaurement and theoretical prediction confirms the parameter value.

As discussed in the main text, we use the same value for the static and the dynamic drop-substrate capacitance. While effects of contact angle hysteresis can substantially change the shape of sliding drops, the wetted area stays approximately constant.[6] Additionally, electrowetting effects might influence the drop capacitance at high drop potentials. For a similar setup of a sliding drop on a 1 mm substrate, Li and Ratschow et al.[7] reported a contact angle decrease due to electrowetting of around 10°. With an initial contact angle of 98°, this would increase the drop capacitance by by a maximum of 17%.
This analysis assumes no interaction between the effects of electrowetting and contact angle hysteresis. Such interactions could potentially deform the wetted area in a way that is not easily described theoretically. Due to this complexity and the low influence suggested by the good agreement of our measurements with reported values, we neglect changes in the drop capacitance.

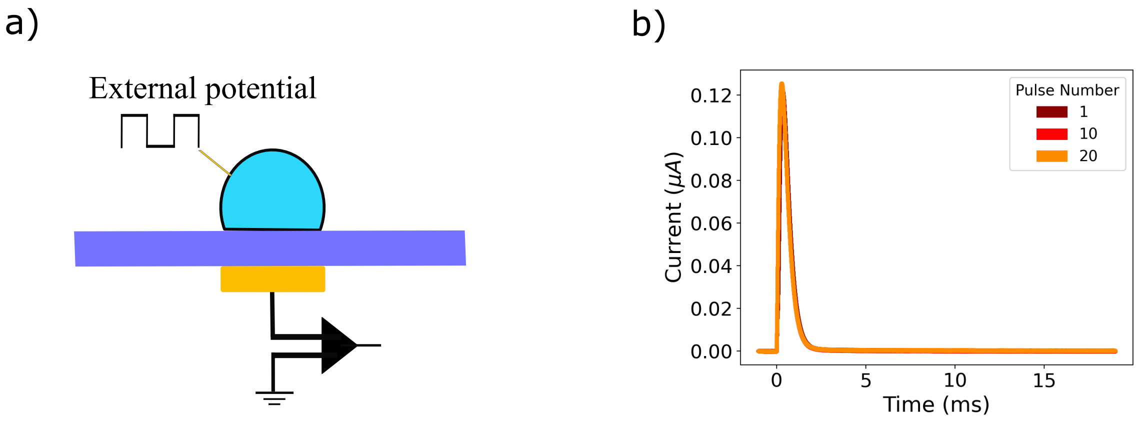

Figure S3: (a) Setup to measure the capacitance using the plate capacitor system. (b) Capacitive current caused by the 0-100V square potential.)

S2 Slide Electrification Theory and Simulation

S2.1 Numerical simulation

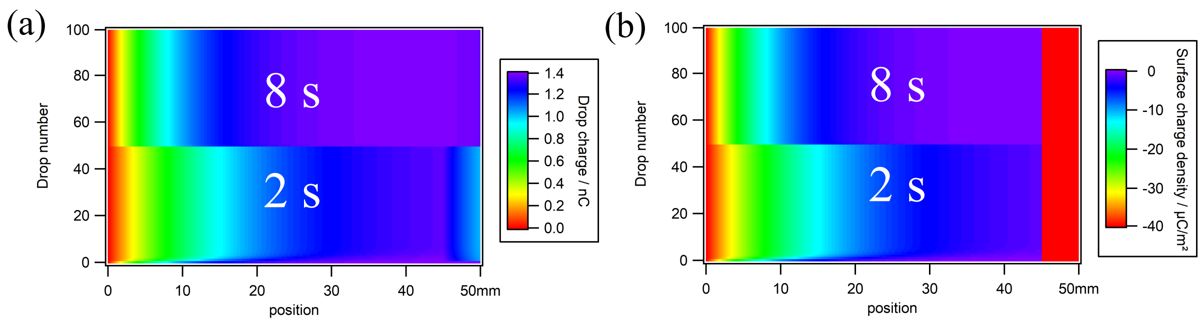

Figure S4: (a) Color plot drop charge, (b) color plot surface charge density generated using numerical simulation.

Both previous slide electrification models[5] and our new model fundamentally consist of a set of coupled differential equations for drop and surface charge. Based on these models, a set of analytical solutions for the first and steady-state drops can be derived.[5] Due to the recursive nature of the equations

$$dQ^n = \left[-\alpha c_s \left[\chi\Phi + U_D(x)\right] + \sigma_{\text{out}}^{n-1}(x) exp(-\Delta t/\tau)\right] wdx \qquad \text{(S2)}$$

$$\sigma^n(x) = \sigma^n_{out}(x), \tag{S3}$$

analytical solutions for every drop in a series of drops are difficult to calculate and yield complicated expressions. Furthermore, experiments with varying drop rate are impossible to simulate this way. Therefore, we wrote a simulation program in Igor Pro (wavemetrics) that solves equations S2 and S3 numerically, generating data sets of drop- and surface charge as a function of distance and drop number. An example of a simulation for a PFOTS surface is shown in figure S4. The figure shows the change in drop and surface charge over the slide length for different times between drops Δt and drop numbers. Within this simulation, we also take into account that the drop is discharged, as soon as it touches the measurement electrode. At this point in time, the receding contact line is located at a distance w before the electrode and starts to deposit the maximum amount of charges, as the drop is now discharged (red region on the right in Figure S4 (b)). The advancing contact line of the following drop will pass this region of maximum surface charge before touching the electrode. This additional σ_{in} contribution reduces the drop charge before the electrode contact, modifying the measured drop charge.

S2.2 Estimation of surface discharge time τ

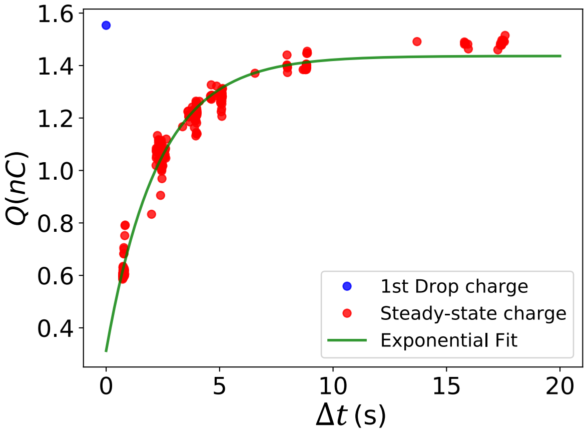

Figure S5: Steady-state drop charge (Q) vs. drop interval (Δt), and fit an exponential fit to obtain decay time.

The gradual charging and discharging of the hydrophobic surface by a succession of drops leads to less charging for later drops. A way to get the discharge time scales is to use steady sate charge vs. drop rate Δt. With increasing time between drops, the steady state charge increases until a limit is reached where the surface charge dissipates completely between drops. This discharge time could be obtained using an equation $Q(\Delta t) = a(1 - e^{\frac{-\Delta t}{\tau}}) + b$ assuming the discharge is an exponential process. Here, we measured the steady-state drop charge measurement vs, Δt at slide length 4 cm, where saturation should be already reached (Fig. S6). From the fit, we obtained the time scales $\tau_c = 2.3 \pm 0.2$ s.

S2.3 Surface potential at different humidity

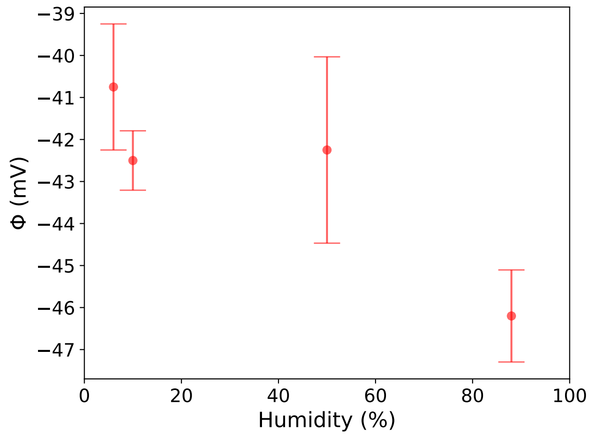

Figure S6: Surface potential Φ with increasing humidity estimated using measured drop potential U_{max} and $\lambda_D = 400nm$.

We measured the maximum drop potential and estimated the surface potential using eq. 7 from the main text. We only observed a slight increase in surface potential at higher humidity. Further systematic and quantitative study is needed to address this slight variation, which is outside the scope of this study

References

(1) Sosa, M. D.; Ricci, M. L. M.; Missoni, L. L.; Murgida, D. H.; Cánneva, A.; D'Accorso, N. B.; Negri, R. M. Liquid–polymer triboelectricity: chemical mechanisms in the contact electrification process. *Soft Matter* **2020**, *16*, 7040–7051.

(2) Diaz, D. I.; González, D. G.; Bista, P.; Weber, S.; Butt, H.-J.; Stetten, A. Z.; Kappl, M. Charging of Impacting Drops onto Superhydrophobic Surfaces. *Soft Matter* **2022**,

(3) Kowacz, M.; Pollack, G. H. Moving Water Droplets: The Role of Atmospheric CO2 and Incident Radiant Energy in Charge Separation at the Air–Water Interface. *The Journal of Physical Chemistry B* **2019**, *123*, 11003–11013.

(4) Vogel, P.; Möller, N.; Qaisrani, M. N.; Bista, P.; Weber, S. A.; Butt, H.-J.; Liebchen, B.; Sulpizi, M.; Palberg, T. Charging of Dielectric Surfaces in Contact with Aqueous Electrolytes- the Influence of CO_2. *Journal of the American Chemical Society* **2022**, *144*, 21080–21087.

(5) Stetten, A. Z.; Golovko, D. S.; Weber, S. A.; Butt, H.-J. Slide electrification: charging of surfaces by moving water drops. *Soft matter* **2019**, *15*, 8667–8679.

(6) Li, X.; Bodziony, F.; Yin, M.; Marschall, H.; Berger, R.; Butt, H.-J. Kinetic drop friction. *Nature Communications* **2023**, *14*, 4571.

(7) Li, X.; Ratschow, A. D.; Hardt, S.; Butt, H.-J. Surface Charge Deposition by Moving Drops Reduces Contact Angles. *Phys. Rev. Lett.* **2023**, *131*, 228201.
