# Supplementary material for: High Voltages in Sliding Water Drops
Source: J Phys Chem Lett. 2023 Dec 5;14(49):11110–6. doi: 10.1021/acs.jpclett.3c02864 (PMC10726385; doi:10.1021/acs.jpclett.3c02864)
Supplement: Supplementary file 2 — jz3c02864_si_002.pdf [file jz3c02864_si_002.pdf]

jz-2023-028643.R1

Name: Peer Review Information for "High Voltages in Sliding Water Drops"

First Round of Reviewer Comments

Reviewer: 1

Comments to the Author

The manuscript by P. Brista, A. Ratschow, et all reports the experiments on the sliding electrification of water droplets on the modified soda-lime glass substrate. The authors discovered the existence of a saturation voltage that is directly proportional to the solid-liquid surface potential difference. They propose the use of the drop experiment as a cost-effective method for measuring the surface potential and give some insights into the mechanism of electrification.

This is high-quality research that provides additional insight into the mechanism(s) of contact electrification. The manuscript is clearly written and meets the high editorial standards of JPCL. There are a few issues that need to be addressed before publication:

1. How does moisture affect the measured voltage? I wonder if the inexpensive surface potential measurements are sensitive to the pre-adsorbed water.

2. Apart from ref. [39] of the manuscript, there are other ideas about the physical mechanism and the theory of contact electrification. For example, in DOI: 10.1021/acs.jpclett.3c00479 the authors discuss the static effect and the saturation value $\varphi 0 \approx 50$ mV, which is close to $|\Phi| = 44$ mV obtained in the current manuscript. Can the authors correlate their model with the existing one that seems to complement their study?

3. The authors mentioned the Debye length as a proportionality factor between charge and surface potential. However, pH can also affect the surface potential and the amount of adsorbed charge, even if the Debye length remains the same. Can the authors comment on this?

4. Previous studies report < 1% efficiency in the conversion of the kinetic energy of the sliding droplets into electrical energy (see e.g. DOI: 10.1016/j.apenergy.2021.117394). Can the authors' theory suggest how to improve efficiency?

5. In the measurements with the capacitive voltage divider, how was the contact potential difference between the Au electrode and water accounted for? As far as I understand, the charge carriers in the water will form an EDL at the gold surface too. If this is a negligible effect, please explain.

6. In the geometry shown in Fig. 1 of the manuscript, the drop can acquire a charge as it separates from the capillary, even if the capillary is grounded. Some of this charge may remain on the glass surface even when the drop is later discharged by contacting the reference electrode. Is this accounted for and how does it affect the conclusions?

7. The Debye length λ_D on page 6, line 15 is defined as the width of the diffuse layer h. I think they are roughly the same, but the original definition of λ_D is different. Perhaps a rephrasing should be considered.

8. The model in Figure 4 is not as perfect as it could be. Can the authors explain why?

9. The estimated surface discharge time τ is about 2.3 s (see Page S6). What could cause such a high value?

Minor remarks:

1. Check the wording on the line 53, page 2, main text.

2. The second half of the capture of Fig. 3 is a discussion. You may want to consider moving this information to the main text.

Reviewer: 2

Comments to the Author

In the manuscript entitled "High voltages in sliding water drops", Bista et al. provide a fundamental understanding of the high voltages generation in sliding water drops on hydrophobic substrates. The experimental data and theoretical analysis are detailed and elaborated. The manuscript can be published in The Journal of Physical Chemistry Letters after the authors address the following comments.

The authors implemented two special experiments to accurately measure the sliding-induced voltage of droplets. However, there are a few concerns that need to be addressed. In the first experiment, it is recommended that the authors provide additional descriptions regarding the configuration of the droplet-substrate capacitors. This could include clarifying what can be considered as conducting sheets, how and where the charge is stored, and whether it is a single capacitor or an equivalent capacitor of two/three capacitors in series. Additionally, further explanation is needed to justify the rationality of using the static droplet-substrate capacitance as a substitute for the dynamic droplet-substrate capacitance in voltage calculations. The authors might want to consider the potential impact of contact angle hysteresis on the shape of the sliding droplets, which may affect the capacitance of the droplet-substrate capacitor. Furthermore, relevant discussions about Fig. 2a are missing in the main text and should be included. In the second experiment, the authors claimed that they used the capacity ratio of

Cin/CD to estimate the initial voltage. However, it is not clear how the value of CD and the capacity ratio are determined. Besides, the authors should provide further explanation or references to support the statement "It is followed by a linear voltage increase due to the ongoing charge separation at the moving contact line".

Another significant finding of the manuscript is the establishment of a general model to describe the high-voltage generation behavior of sliding droplets. The authors simplify the capacitance of the electric double layer (EDL) to the capacitance of the diffusion layer and then connect it in series with the substrate capacitor for model building and calculation. However, this simplification is strongly influenced by surface potentials and ion concentration. Though the authors provide a factor, log(c), for correction, it is important for the authors to provide a more specific boundary condition for their model and clarify whether the correction factor, log(c), is always applicable. Besides, the authors should also check the statement "It is well known that at low potentials or high ion concentration, Φ and λD are approximately proportional to √c, making the product Φ/λD independent of c", on page 10, line 5. I think it should be "low ion concentration".

My other concern is about the charge transfer coefficient. In the manuscript, the authors link the surface potential and droplet voltage with a very simple but accurate model, which, however, lacks a detailed description of how the surface and the droplet are charged. Several investigations, as documented in DOI:10.1002/dro2.22, have elucidated strategies for harnessing the intricate interplay between water and electricity, which may serve as valuable points of reference to improve the discussion. It would be also beneficial if the authors could provide a model or description explaining this process and identify the parameters that influence the charge transfer coefficient.

Energy conversion efficiency is also very important for energy applications. It would be valuable to discuss or provide references to relevant models of energy conversion of droplets' electrostatic energy.

Author's Response to Peer Review Comments:

First of all, we would like to thank referees for their overall positive and constructive feedback. We tried to implement all the recommendations and answer all open questions regarding our manuscript.

Referees Response

Recommendation: This paper is probably publishable, but major revision is needed; I do not need to see future revisions.

Reviewer: 1

Comments:
The manuscript by P. Bista, A. Ratschow, et all reports the experiments on the sliding electrification of water droplets on the modified soda-lime glass substrate. The authors discovered the existence of a saturation voltage that is directly proportional to the solid-liquid surface potential difference. They propose the use of the drop experiment as a cost-effective method for measuring the surface potential and give some insights into the mechanism of electrification.

This is high-quality research that provides additional insight into the mechanism(s) of contact electrification. The manuscript is clearly written and meets the high editorial standards of JPCL. There are a few issues that need to be addressed before publication:

1. How does moisture affect the measured voltage? I wonder if the inexpensive surface potential measurements are sensitive to the pre-adsorbed water.

Thank you for your question. Indeed, the study of voltage at different humidity could be quite interesting. We conducted a couple of preliminary experiments at different relative humidities from 5% to 88%. We measured a small shift from -40 ± 1 mV for 5% humidity to -46 ± 1 mV for 88% humidity, so the values obtained are quite consistent over a wide range of humidities within our experimental error. Nevertheless, we think a systematic and quantitative study is needed to address this question, which is outside the scope of this study.

We nevertheless added the measurements with a short discussion to the supporting information in SI line 132 and added: “No significant difference in measured voltage was observed between 5% to 88% humidity.” in line 135 in the main manuscript.

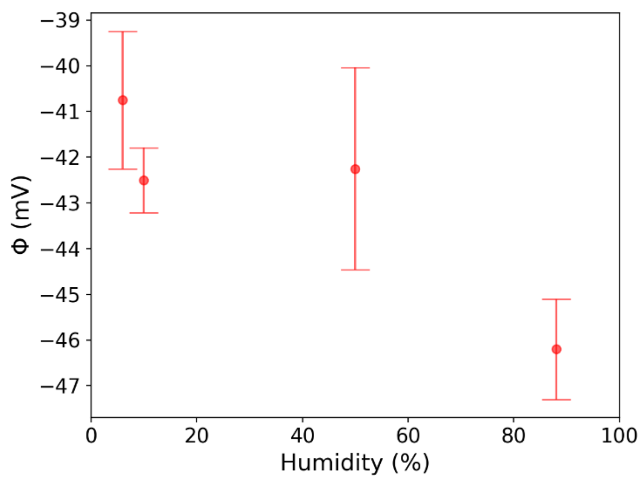

2. Apart from ref. [39] of the manuscript, there are other ideas about the physical mechanism and the theory of contact electrification. For example, in DOI: 10.1021/acs.jpclett.3c00479 the authors discuss the static effect and the saturation value $\varphi 0 \approx 50$ mV, which is close to $|\Phi| = 44$ mV obtained in the current manuscript. Can the authors correlate their model with the existing one that seems to complement their study?

We thank the referee for pointing us at this interesting paper. The setup for the model is quite different from the present case. Artemov et al. discuss a situation where charges redistribute between the solid-liquid and the liquid-gas interface, while the solid-gas interface remains charge free. Still, their theory and physical mechanism also revolve around the surface potential of the solid-liquid interface and thus supports our own claims. We have added the paper to the bibliography as ref. [35].

3. The authors mentioned the Debye length as a proportionality factor between charge and surface potential. However, pH can also affect the surface potential and the amount of adsorbed charge, even if the Debye length remains the same. Can the authors comment on this?

The referee rightfully pointed out that changes in pH can also change the surface potential at constant Debye lengths. To our knowledge, this is caused by specific ion adsorption and there is no universal model for this effect - only approximations under certain assumptions or phenomenological fitting models (cf. refs. [35, 38, 40, 42, 43, 45, 46]). In the light of this lack of understanding, the procedure introduced in our manuscript could provide new perspectives, as it allows a simple and easy measurement of the pH-induced changes in surface potential.

4. Previous studies report < 1% efficiency in the conversion of the kinetic energy of the sliding droplets into electrical energy (see e.g. DOI: 10.1016/j.apenergy.2021.117394). Can the authors' theory suggest how to improve efficiency?

We thank the referee for this valuable suggestion. Our findings indeed have implications for energy harvesting. The theoretical analysis reveals that the saturation voltage increases linearly with substrate thickness, while the saturation charge and saturation length scale are independent of substrate thickness. Thus, the energy harvesting efficiency can be increased by choosing a thicker substrate. Further, for series of drops, the saturation voltage decreases due to surface charge left by previous drops. Thus, substrate materials with a faster decay of surface charge allow for higher energy conversion efficiency.
We addressed this by adding the following sentences to the text, starting from line 181:
"Our findings have immediate implications for energy harvesting from sliding drops. Previous studies reported rather low efficiencies of around 1%. [23,50] Our theoretical analysis reveals that the saturation voltage $U_{max,D}$ increases proportionally to the substrate thickness (eq. 7), while the saturation charge Q only depends on the wetted area and the EDL properties (eq. 9). Thus, the total drop energy $W_D = 1/2QU_D$ increases linearly with the substrate thickness. In the present case, the drop charge saturates after sliding a height difference of $\Delta z = 3$ cm, losing a potential energy of $W_g = mg\Delta z = 13\ \mu J$. The electrical energy on a 1 mm thick substrate is f $W_D = 0.8 \mu J$, yielding an energy harvesting efficiency of 6%. By increasing the substrate thickness to 3 mm, the efficiency increases to 18%. Here, the upper limit is that the drops get stuck from their own electrostatic field. Thus, to optimize this process, a balance between droplet motion and energy harvesting has to be found."
And in line 198:
"For subsequent drops, the saturation length increased due to the fact that the surface was already partially charged from previous drops, reducing the charge transfer."

5. In the measurements with the capacitive voltage divider, how was the contact potential difference between the Au electrode and water accounted for? As far as I understand, the charge carriers in the water will form an EDL at the gold surface too. If this is a negligible effect, please explain.

The electrode that we use to probe the charged drop is grounded before the measurement. The potential difference between the electrode and the charged drop (~kV) is much greater than the contact potential difference between the metal electrode and the water (<0.1V), so we can easily neglect this effect.

6. In the geometry shown in Fig. 1 of the manuscript, the drop can acquire a charge as it separates from the capillary, even if the capillary is grounded. Some of this charge may remain on the glass surface even when the drop is later discharged by contacting the reference electrode. Is this accounted for and how does it affect the conclusions?

We agree with he referee that there are undefined charging processes happening during the drop deposition, both at the capillary and at the place where the drop impacts the substrate. This is exactly the reason why we added a grounding electrode at the beginning of the sliding path. The position where the drop leaves this electrode marks the position zero for our slide electrification experiments. From here on, there is only a weak electrostatic interaction between the now neutral drop and the static charges at the position of impact.

For our 2022 Nature Physics Paper, we conducted extensive simulations on the electrostatic forces on the drop (Ref 14). We recently extended these simulations to include the counter electrode under the substrate and potential impact charges in the region before the grounding electrode x < 0 cm (see graph below). If we include an impact charge on the order the saturation charge of the first drop (1 nC) in the region 2 cm ahead of the grounding electrode, we see no deviations in the electrostatic force. Only when including a 100 times higher impact charge, we can generate a considerable amount of electrostatic force within the first 1-2 cm of the drop path.

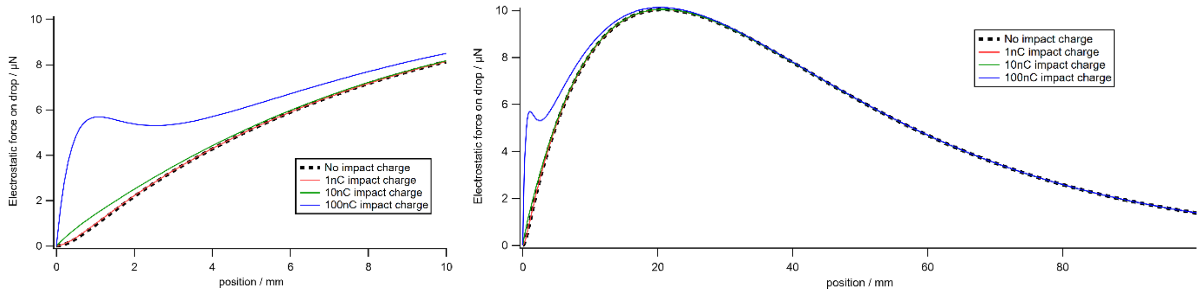

Regardless of the implications on the electrostatic force, the saturation charge and voltage will be measured after more than 2-3 cm, where the effect of the impact charges – even if they are unexpectedly high – can be neglected.

7. The Debye length λ_D on page 6, line 15 is defined as the width of the diffuse layer h. I think they are roughly the same, but the original definition of λ_D is different. Perhaps a rephrasing should be considered.
We rephrased the sentence to "The characteristic thickness of this diffuse layer is called the Debye length λ_D." in line 108.

8. The model in Figure 4 is not as perfect as it could be. Can the authors explain why?

Thank you for the excellent question. We currently think that the deviations originate in slow polarization effects within the substrate. Substrate polarization can happen in float glass because of ion migration within the strong electric fields between the drop and the counter electrode (see also the answer to the next question). We are working on a theory to describe these deviations. For the current manuscript we decided not to include these corrections as it would go beyond the scope of the current study.

We addressed this by adding, “Deviations from the model might be explained by change in velocity, either caused by electrostatic forces between the drop and surface charges (Ref. 14). Furthermore, the observed behavior might be caused by substrate polarization” in line 204 in the main manuscript.

9. The estimated surface discharge time τ is about 2.3 s (see Page S6). What could cause such a high value?

The surface discharge time depends on two factors: the resistivity of the substrate (glass) and neutralization through atmospheric ions.

To measure the substrate resistivity, we applied a high voltage of 200 V for 10 s between a drop and the electrode under the substrate and measured a transient capacitive current that saturated to a finite value of $8.8 \cdot 10^{-11}$A, yielding a resistivity of 2.3 $T\Omega$ (see graph below). If we consider this value and the capacitance of the substrate under the drop to be $1.2 \cdot 10^{-12}$F, we calculate the discharge time to be approximately 2 seconds, which is close to the value we measured. We are planning on another manuscript discussing the discharge process in detail.

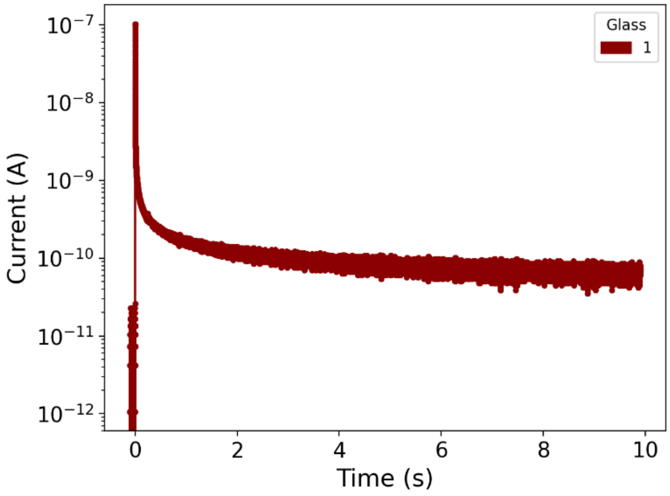

Minor remarks:
1. Check the wording on the line 53, page 2, main text.
2. The second half of the capture of Fig. 3 is a discussion. You may want to consider moving this information to the main text.

Thank you for the recommendation. Your suggestion has been valuable to improve our manuscript.

Reviewer: 2

Recommendation: This paper may be publishable, but major revision is needed; I would like to be invited to review any future revision.

Comments:
In the manuscript entitled "High voltages in sliding water drops", Bista et al. provide a fundamental understanding of the high voltages generation in sliding water drops on hydrophobic substrates. The experimental data and theoretical analysis are detailed and elaborated. The manuscript can be published in The Journal of Physical Chemistry Letters after the authors address the following comments.

The authors implemented two special experiments to accurately measure the sliding-induced voltage of droplets. However, there are a few concerns that need to be addressed. In the first experiment, it is recommended that the authors provide additional descriptions regarding the configuration of the droplet-substrate capacitors. This could include clarifying what can be considered as conducting sheets, how and where the charge is stored, and whether it is a single capacitor or an equivalent capacitor of two/three capacitors in series.

Thank you for your question. We considered the metal plate underneath the glass as the bottom plate of the capacitor and the PFOTS-water interface within the drop as the top plate of the substrate capacitor. To minimize the electrostatic energy, the charges accumulating in the drop during sliding will accumulate as close as possible to the grounded counter electrode under the substrate, i.e., at the PFOTS-water interface. The net surface charge will be the sum of this accumulated charge and the native surface charge density due to the surface/zeta potential.

We totally agree that there are multiple capacitors in the system. However, due to the significantly smaller capacitance of the substrate compared to the double layer capacitance, the total capacitance in series is primarily determined by the smaller capacitance. For instance, if we consider a double layer capacitance of 10μF [ref. 38] and a glass substrate capacitance of 1 pF in series, the total capacitance is approximately 1 pF. Therefore, for simplification, we treat the equivalent capacitor as the substrate capacitor.

We addressed this by adding, “which is the equivalent capacitance of the drop-substrate system.” in line 50 in the main manuscript.

Additionally, further explanation is needed to justify the rationality of using the static droplet-substrate capacitance as a substitute for the dynamic droplet-substrate capacitance in voltage calculations. The authors might want to consider the potential impact of contact angle hysteresis on the shape of the sliding droplets, which may affect the capacitance of the droplet-substrate capacitor.

We agree that contact angle hysteresis can substantially change the shape of sliding drops. For the droplet-substrate-capacitance, the total wetted area is the crucial parameter. Fig 4d in DOI: 10.1038/s41467-023-40289-8 clearly shows that while the drop elongates, its width also decreases at high velocities. Nevertheless, the approximately elliptical footprint area of $A = \pi \, l \, w$ stays roughly constant. Thus, the influence of contact angle hysteresis alone is – in good approximation – negligible. A second mechanism that can increase the wetted area in sliding drops is electrowetting.

We added the following discussion to the SI:
“As discussed in the main text, we use the same value for the static and the dynamic drop-substrate

capacitance. While effects of contact angle hysteresis can substantially change the shape of sliding drops, the wetted area stays approximately constant. [6] Additionally, electrowetting effects might influence the drop capacitance at high drop potentials. For a similar setup of a sliding drop on a 1 mm substrate, Li and Ratschow et al. [7] reported a contact angle decrease due to electrowetting of around 10°. With an initial contact angle of 98°, this would increase the drop capacitance by a maximum of 17%. This analysis assumes no interaction between the effects of electrowetting and contact angle hysteresis. Such interactions could potentially deform the wetted area in a way that is not easily described theoretically. Due to this complexity and the low influence suggested by the good agreement of our measurements with reported values, we neglect changes in the drop capacitance."

Furthermore, relevant discussions about Fig. 2a are missing in the main text and should be included.

Thank you for the suggestion and question.

To address your initial concern, we included additional information in the manuscript. In line 66, we added the following: "We measured drop charge and voltage at different slide lengths. The drop charge and voltage values as a function of slide length for different drops are shown in figure 2a, which we refer to as drop charge traces." In line 198, we explain, "For subsequent drops, the saturation length increased due to the surface already being partially charged from previous drops, which reduced the charge transfer."

In the second experiment, the authors claimed that they used the capacity ratio of Cin/CD to estimate the initial voltage. However, it is not clear how the value of CD and the capacity ratio are determined.

We determined the capacitance of the input capacitor using a known resistance of 400 MΩ and the discharge time from the fit, as explained in Supplementary Material S1.3. To estimate the capacitance of C_D, we employed the static drop method as discussed in S1.4. Additionally, we used a plate capacitor model estimation and the values agreed well with our measured values. Another method to verify C_D and U_D involves modifying the input capacitor, C_{in}. By reducing the input capacitor value from 1350 pF to 300 pF, we were able to observe an increase in voltage from approximately 1 V to 4 V. In this experiment, C_D and U_D should remain constant.

Nevertheless, you are right, the ratio is not completely clear from our current description. Therefore, we added the following in line 92 of the main text: "$(C_{in} + C_D)/C_D \approx C_{in}/C_D$ for C_{in} >> CD".

We also added the following in line 61 in the Supplementary Information (SI):" The voltage in capacitor C_D before the contact with the probe capacitor, with capacitance C_{in}, is denoted as U_D. The charge in C_D is represented as Q. After the contact, the total charge equilibrates between the two parallel capacitors, and both attain the same voltage. The total charge in this situation is given by $Q = C_{total} U_{in} = (C_D + C_{in}) U_{in}$. Solving this equation yields the voltage ratio: $U_D = C_{in} + \frac{C_{in}+C_D}{C_D} U_{in}$."

Besides, the authors should provide further explanation or references to support the statement "It is followed by a linear voltage increase due to the ongoing charge separation at the moving contact line".

In our recent study, as referenced in [40] in the main manuscript, we thoroughly discussed the charge separation at the moving contact line and its parametric dependencies. To measure the charge separation at the moving contact line, we monitored the current as a grounded drop slid on the surface. The linear voltage increase observed in this study corresponds to the current due to ongoing charge

separation at the receding contact line, which can be estimated as follows.

The charge separation at the receding contact line results in a current (I), which can be estimated as $I = C_{in}\frac{dV}{dt}$,where $\frac{dV}{dt}$ represents the slope of voltage vs. time. By estimating this current using C_{in}, we obtained a value of I = (70-80) nA. Notably, this value aligns with the measurements we reported in [40].

We added the following in line 87 to address this "The current resulting from ongoing charge separation can be estimated as $I = C_{in}\frac{dV}{dt}$, which yields a value of 70-80 nA. This estimation closely matches the values measured in a previous study [Refs. 40] and can also be seen in the drop discharge current in Figure S1c."

Another significant finding of the manuscript is the establishment of a general model to describe the high-voltage generation behavior of sliding droplets. The authors simplify the capacitance of the electric double layer (EDL) to the capacitance of the diffusion layer and then connect it in series with the substrate capacitor for model building and calculation. However, this simplification is strongly influenced by surface potentials and ion concentration.

In our model, we show how the high voltages in drops can be explained by the surface potential and the capacitance ratio. The model can thus explain and predict drop charging for known ion concentrations and surface potentials. Changing the ion concentration, in addition to changing the Debye length, can additionally affect the surface potential. We did not go into the details of surface potential dependence on salt concentration in this manuscript as it is a very general problem in physical chemistry and falls outside the scope of our current study.

Though the authors provide a factor, log(c), for correction, it is important for the authors to provide a more specific boundary condition for their model and clarify whether the correction factor, log(c), is always applicable. Besides, the authors should also check the statement "It is well known that at low potentials or high ion concentration, Φ and λD are approximately proportional to √c, making the product Φ/λD independent of c", on page 10, line 5. I think it should be "low ion concentration".

Building on our answer to the previous question, we acknowledge the importance of exploring this aspect in future research. Our primary aim was to demonstrate that our measured potentials as function of ion concentration scale in a similar way to established theories and previous measurements [Ref. 48]. The behavior of surface potential with increasing salt concentration in our study spans two distinct regimes, and a single analytical model cannot fully describe it. As discussed in reference 49, we observe two different regimes: for low potential, it scales with $c^{-\frac{1}{2}}$, and for higher potential, it scales with $\log(c)$). The scaling with log(c) effectively describes a wide range of concentrations, which is why we applied it in our observations. However, it is worth noting that this model does not fully capture the behavior in the high concentration regime. Nonetheless, it serves to illustrate that the voltage we measured exhibits similar scaling to the surface potential.

Most importantly, our interpretation of the saturation drop voltage enables a cheap and simple experimental method to study these dependencies of surface potentials on ion concentration. The only input parameters of the model are substrate- and solution properties with the ion concentration providing the characteristic width of the Debye layer. Thus, our method can provide new data for the refinement of models describing the complex processes determining the surface potential.

My other concern is about the charge transfer coefficient. In the manuscript, the authors link the surface potential and droplet voltage with a very simple but accurate model, which, however, lacks a detailed description of how the surface and the droplet are charged. Several investigations, as documented in DOI:10.1002/dro2.22, have elucidated strategies for harnessing the intricate interplay between water and electricity, which may serve as valuable points of reference to improve the discussion. It would be also beneficial if the authors could provide a model or description explaining this process and identify the parameters that influence the charge transfer coefficient.

Thank you for pointing out the literature DOI:10.1002/dro2.22. We added it in our discussion. One important result of our theoretical model is that the saturation voltage is independent of the charge transfer coefficient, which only determines the sliding length until saturation. Even if there is a somehow time-, distance- or velocity-dependent charge transfer coefficient, the saturation voltage would still be the same. In fact, in our recent study, 'How charges separate when surfaces are dewetted' [Ref. 40], we conducted a comprehensive investigation, using theoretical, numerical, and experimental methods to explore the impact of various parameters, such as contact angle, velocity, and more. As an important result, we find that the charge transfer coefficient is almost constant in the velocity regime relevant for this work.

We added the following in line 113 to address this ", as valid for the drop velocity range during the experiments,"

Energy conversion efficiency is also very important for energy applications. It would be valuable to discuss or provide references to relevant models of energy conversion of droplets' electrostatic energy.

We thank you for pointing this out. In fact, our theoretical analysis shows that the saturation voltage increases linearly with substrate thickness, while the saturation charge and saturation length scale are independent of substrate thickness. Thus, the efficiency of energy harvesting can be improved by choosing a thicker substrate. Further, for series of drops the saturation voltage drops due to surface charge left by previous drops. Thus, substrate materials with a faster decay of surface charge allow for higher energy conversion efficiency. We believe that these findings significantly improved the paper and addressed them by adding the following sentences to the text:

"Our findings have immediate implications for energy harvesting from sliding drops. Previous studies reported rather low efficiencies of around 1%. [23,50] Our theoretical analysis reveals that the saturation voltage $U_{max,D}$ increases proportionally to the substrate thickness (eq. 7), while the saturation charge Q only depends on the wetted area and the EDL properties (eq. 9). Thus, the total drop energy W_D = $1/2QU_D$ increases linearly with the substrate thickness. In the present case, the drop charge saturates after sliding a height difference of Δz = 3 cm, losing a potential energy of $W_g = mg\Delta z = 13\ \mu J$. The electrical energy on a 1 mm thick substrate is f $W_D = 0.8 \mu J$, yielding an energy harvesting efficiency of 6%. By increasing the substrate thickness to 3 mm, the efficiency increases to 18%. Here, the upper limit is that the drops get stuck from their own electrostatic field. Thus, to optimize this process, a balance between droplet motion and energy harvesting has to be found."
And in line 198: "For subsequent drops, the saturation length increased due to the fact that the surface was already partially charged from previous drops, reducing the charge transfer."

We thank the referees for the questions raised that helped us to significantly clarify and improve our manuscript.

Editor Suggestion:
1) Title: In both the main manuscript file and the Supporting Information, set the title in title case, with the first letter of each principal word capitalized.
Thank you, we adapted the suggestion in the manuscript.

2) Figures: Remove "Part A" from figure caption of Figure 4 if it does not have different parts.
3) References: In both the main file and the supporting information, fix the style of all references to use JPCL formatting (check all references carefully). ***JPC Letters reference formatting requires that journal references should contain: () around numbers; author names; article title (titles entirely in title case or entirely in lower case); abbreviated journal title (italicized); year (bolded); volume (italicized); and pages (first-last). Book references should contain author names; book title (in the same pattern); publisher; city; and year. Websites must include date of access.
Thank you, we adapted the suggestion in the manuscript.
4) TOC Graphic: Please resize the TOC graphic per journal guidelines (2 in x 2 in) and move to the correct position (on the same page as the abstract).
Thank you, we adapted the suggestion in the manuscript.

5) Supporting Information Statement: The supporting-information (SI) paragraph in the main file is incomplete. Please list each item (including graphics) that can be found in the SI-for-Publication file individually.
*Examples of sufficient descriptions: "Supporting Information: 1H NMR spectra for all compounds" or "Additional experimental details, materials, and methods, including photographs of experimental setup".
*Examples of insufficient descriptions: "Supporting Information: Figures S1-S3" or "Additional figures as mentioned in the text".
Thank you, we adapted the suggestion in the manuscript.
